# Sirt1 and Sirt6 Mediate Beneficial Effects of Rosiglitazone on Hepatic Lipid Accumulation

**DOI:** 10.1371/journal.pone.0105456

**Published:** 2014-08-18

**Authors:** Soo Jin Yang, Jung Mook Choi, Eugene Chang, Sung Woo Park, Cheol-Young Park

**Affiliations:** 1 Department of Food and Nutrition and Human Ecology Research Institute, Chonnam National University, Gwangju, Korea; 2 Diabetes Research Institute, Sungkyunkwan University School of Medicine, Kangbuk Samsung Hospital, Seoul, Korea; 3 Department of Endocrinology and Metabolism, Sungkyunkwan University School of Medicine, Kangbuk Samsung Hospital, Seoul, Korea; University of Hong Kong, China

## Abstract

**Background:**

Sirtuin (Sirt), a sensor of the cell metabolic state, regulates glucose and lipid metabolism. The aim of this study was to address whether rosiglitazone (RGZ) alters hepatic Sirt1 and whether Sirt1 and/or Sirt6 have a regulatory role in the protective effects of RGZ on hepatocyte steatosis.

**Methods:**

To investigate the effect of RGZ on hepatic Sirt1, rats were administered with RGZ for 6 weeks. The involvement of Sirt1/6 in the RGZ-mediated effect against hepatic steatosis was evaluated by single or double knockdown of Sirt1 and Sirt6 in a hepatocyte steatosis model.

**Results:**

RGZ *in vivo* increased Sirt1 expression and its activity in rat livers. In a hepatocyte steatosis model, RGZ significantly reduced lipid accumulation and activated the Sirt1/6-LKB1-AMPK pathway. Sirt1 knockdown abolished the effects of RGZ with regard to hepatocyte fat accumulation and the Sirt1/6-LKB1-AMPK pathway, suggesting that Sirt1 is a key regulator of RGZ-mediated metabolic processes. Sirt6 knockdown inhibited the protective effects of RGZ to a lesser extent than Sirt1, and double knockdown of Sirt1/6 showed no synergistic effects.

**Conclusion:**

Our results demonstrate that Sirt1/6 are involved in the RGZ-mediated effects on hepatocyte steatosis, and the regulatory effects of Sirt1 and Sirt6 are not synergistic but compensatory for improving hepatocyte steatosis.

## Introduction

Sirtuin (Sirt) has been considered as a metabolic sensor to control glucose and lipid metabolism; therefore, dysfunction of its pathway results in the development of diabetes and hepatic steatosis [1]–[4]. Seven mammalian isoforms of sirtuins, which differ in location and biological functions, were identified [5], [6]. Sirt1 and Sirt6 have been intensively investigated in the context of metabolic regulation. Sirt1 transgenic mice exhibit reduced levels of fasting blood glucose and insulin as well as improved glycemic control, showing anti-diabetic effects, during the glucose tolerance test [7], [8]. Additionally, Sirt1 overexpression in mice protects against hepatic steatosis induced through a high-fat diet [9]. However, Sirt1 deficiency in mice leads to hepatic steatosis and inflammation [10], and liver-specific Sirt1 knockout mice develop severe hepatic steatosis and late-onset obesity with impaired whole-body energy expenditure [11]. Sirt6 possesses similarities to Sirt1 in cellular localization and metabolic regulation. Both are localized in the nucleus and are involved in glucose and lipid metabolism. Sirt6 transgenic mice are protected from hepatic fat accumulation and pathological damage due to diet-induced obesity [12], and Sirt6 knockout mice show fatty liver formation and alterations in insulin sensitivity and glucose metabolism [2], [13].

Several pathways have been suggested as the underlying mechanisms of the regulatory effects of Sirt1 and Sirt6, including AMP-activated protein kinase (AMPK), fibroblast growth factor 21, forkhead box O1 (Foxo1), hypoxia-inducible factor 1-alpha, liver kinase B1 (LKB1), and peroxisome proliferator-activated receptor gamma (PPARγ) coactivator-1-α (Ppargc1a/PGC1-α) [11], [14]–[20]. In addition to the regulatory effects of Sirt1 and Sirt6 on diabetes and hepatic steatosis, other sirtuins including Sirt2 and Sirt3 also demonstrate the possibility of acting as metabolic regulators [21]–[23], which suggests that sirtuins’ actions on metabolism seem to be, in part, overlapping and redundant.

Rosiglitazone (RGZ) is a thiazolidinediones (TZD)-class anti-diabetic agent, and its action is through activation of PPARγ, a transcription factor sensitizing insulin action and regulating glucose and lipid metabolism as well as inflammation [24]–[26]. Adiponectin and AMPK have been suggested to be key players in the TZD-mediated metabolic effects [27]–[29]. RGZ significantly increases the release of adiponectin presumably via action on PPARγ, which activates AMPK [29]. In addition to the PPARγ-dependent action, TZD can control metabolic processes via PPARγ-independent mechanisms. In a mouse model of alcoholic fatty liver disease, RGZ treatment activated the hepatic Sirt1-AMPK signaling pathway, resulting in increased fatty acid oxidation and inhibited lipogenesis in the liver [18]. Our previous reports regarding RGZ and hepatic steatosis demonstrated that RGZ-mediated improvement of hepatic steatosis is by activating the Sirt6-AMPK pathway in rats and in AML12 mouse hepatocytes [30]. However, the effects of RGZ on Sirt1 in *in vitro* and *in vivo* models of non-alcoholic fatty liver disease (NAFLD) and the synergistic effects of different sirtuin isoforms on metabolic regulation have not yet been reported.

Based on the previous findings on the involvement of sirtuins-AMPK pathway on TZD’s beneficial effects and the similarities in the cellular localization and metabolic regulation between Sirt1 and Sirt6, we hypothesized that RGZ may activate the Sirt1/6-AMPK pathway and Sirt1 and Sirt6 may exert synergistic effects on the RGZ-mediated actions.

The present study was designed to test whether RGZ alters hepatic Sirt1 and whether Sirt1 and/or Sirt6 have a regulatory role in the protective effects of rosiglitazone (RGZ) on hepatocyte steatosis. To seek further evidence for the potential synergistic effects of Sirt1 and Sirt6 on the regulation of hepatic fat accumulation by RGZ, RNA interference (RNAi)-mediated single or double knockdown of Sirt1 and/or Sirt6 was induced in AML12 mouse hepatocytes.

## Materials and Methods

### Animals

Male Otsuka Long-Evans Tokushima Fatty (OLETF) rats and age-matched Long-Evans Tokushima Otsuka (LETO) rats were provided by Otsuka Pharmaceutical (Tokushima, Japan). The rats were maintained in a temperature- and humidity-controlled room with a 12 h light/dark cycle and fed PicoLab Rodent Diet 20 5053 (5% wt/wt fat; Purina Mills, Richmond, IN, USA) with unlimited access to food and water. To investigate the effect of RGZ on hepatic steatosis, a total of eighteen rats at 32 weeks of age were treated with RGZ (4 mg·kg^−1^·day^−1^; Cayman Chemical, Ann Arbor, MI, USA) or vehicle (PBS) via stomach gavage for 6 weeks. After the treatment period, the rats were anesthetized with intraperitoneal Zoletil/Rompun after an overnight fast, and blood was collected via the abdominal aorta. After the blood collection, tissues were harvested and stored at −80°C until further analysis. The study protocol conformed to the specifications outlined in the National Institutes of Health's Guiding Principles for the Care and Use of Laboratory Animals and was approved by the Institutional Animal Care and Use Committee of the Sungkyunkwan University Kangbuk Samsung Hospital (Approval ID: 201010013).

### Cells and culture conditions

AML12 mouse hepatocytes (American Type Culture Collection, Manassas, VA, USA**)** were cultured in DMEM/F-12 (Invitrogen, Carlsbad, CA, USA) supplemented with 10% fetal bovine serum, antibiotics (100 units/ml penicillin and 100 µg/ml streptomycin), 0.1 µM dexamethasone, and a mixture of insulin, transferrin, and selenium (Invitrogen). Subsets of AML12 hepatocytes were treated with palmitic acid (250 µM; Sigma-Aldrich, St Louis, MO, USA; PA) for 48 h to induce hepatocyte steatosis. Then, PA and/or RGZ (10 µM) were administered for an additional 24 h.

RNAi-mediated gene silencing was performed according to the manufacturer’s instructions. AML12 cells were transfected with negative control siRNA (Stealth RNAi negative control duplexes; Invitrogen) or Stealth RNAi siRNA targeting Sirt1 and/or Sirt6 using Lipofectamine (Invitrogen). After a 24 h transfection, subsets of cells were incubated with PA for 48 h to induce hepatocyte steatosis. Then, cells were cultured with the presence or absence of PA and/or RGZ for additional 24 h.

### Metabolic parameters

Triglyceride (TG) contents were measured with enzymatic assays (Sigma). Free fatty acids (FFA) concentrations in cell extracts were measured with a commercial kit from Wako (Wako Pure Chemical Industries, Osaka, Japan). All values of TG and FFA in cell extracts were normalized to the protein concentration.

### Sirt1 deacetylase activity assay

The Sirt1 activity assay is based on the deacetylation of a synthetic substrate (Sirt1 direct peptide; Cayman Chemical) by Sirt1, consisting of four amino acids with one acetylated lysine group (Arg-His-Lys-Lys(Ac)) and a fluorochrome (7-amino-4-methylcoumarin, AMC). The substrate was incubated with samples along with NAD^+^ as a cosubstrate. After deacetylation, the fluorochrome AMC was specifically released only from the deacetylated substrate. Fluorescent intensity was detected using an excitation wavelength of 355 nm and an emission wavelength of 460 nm. Sirt1 deacetylation activity was expressed as a percentage of control.

### Isolation of total RNA and quantitative reverse transcription-polymerase chain reaction (RT-PCR)

Total RNA was isolated from cells and tissues with a PureLink RNA Mini Kit (Invitrogen). Reverse transcription was performed using a High-Capacity RNA-to-cDNA kit (Applied Biosystems, Foster city, CA, USA) following the manufacturer’s instructions. mRNA expression was quantified via real-time PCR (LightCycler 480 system; Roche, Indianapolis, IN, USA). Synthesized cDNA was mixed with LightCycler 480 Probes Master Mix (Roche) and a gene-specific primer and probe mixture (Universal ProbeLibrary system; UPL; Roche). Individual reactions for target and β-actin or GAPDH were carried out separately with negative controls lacking cDNA. The conditions used were as follows: 95°C for 10 min, followed by 40 cycles of denaturation (95°C for 10 s) and annealing/extension (60°C for 20 s). The cycle number for threshold of detection was determined using LightCycler 480 software (Roche). mRNA expression of each target was normalized to that of the β-actin or GAPDH gene and expressed as fold change relative to controls.

### Western blot analysis

Total protein was isolated from tissues and cells via homogenization in cold RIPA lysis buffer (Amresco, Solon, OH, USA) containing protease and phosphatase inhibitors (Roche). The lysates were centrifuged and supernatants were collected and subjected to Western blot analysis. Protein concentrations were measured using the Bio-Rad protein assay (Bio-Rad Laboratories, Hercules, CA, USA) according to the manufacturer’s instructions. Western blotting was performed by denaturing 50 µg of protein at 95°C for 5 min in Laemmli sample buffer (Fermentas, Burlington, Ontario, Canada). Sample proteins were separated on sodium dodecyl sulfate-polyacrylamide gel electrophoresis and transferred to a polyvinylidene difluoride membrane. The membranes were blocked in 5% nonfat dry milk in Tris-buffered saline/Tween-20 (50 mM Tris, pH 7.5, 500 mM sodium chloride, and 0.05% Tween-20) for 1 h at room temperature. The membranes were incubated overnight at 4°C with primary antibodies for AMPKα, phospho-AMPKα (Thr172), LBK1, LKB1 (Ser428), beta-actin (Cell Signaling Technology, Danvers, MA, USA), and Sirt1 (Abcam, Cambridge, UK). Subsequently, the membranes were exposed to an anti-rabbit secondary antibody conjugated with horseradish peroxidase (Santa Cruz Biotechnology, Santa Cruz, CA, USA) for 1 h at room temperature. Signals were detected by chemiluminescence using the ECL detection reagent (GE Healthcare, Piscataway, NJ, USA). The bands were scanned with a Geliance 600 Imaging System (PerkinElmer, Waltham, MA, USA) with a cooled 12-bit camera, and quantified by densitometry. Levels of phosphorylated AMPK and LKB were normalized to values for AMPK and LKB, respectively.

### Statistical analysis

All statistical analyses were performed using SPSS Statistics 21 (SPSS, Inc., Chicago, IL, USA). Data are expressed as means ± SEM. Student’s *t*-test was performed to compare the two groups. Statistical significance was defined as p<0.05.

## Results

### RGZ up-regulates Sirt1 gene expression and increases Sirt1 deacetylase activity in OLETF rat livers

**Figure 1:**
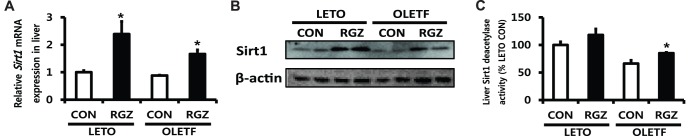
Rosiglitazone (RGZ) alters (A) the gene expression and (B) protein level of Sirt1 as well as (C) Sirt1 deacetylase activity in the livers of control (CON) and RGZ-treated rats. The mRNA expression levels were normalized with respect to those of the glyceraldehyde 3-phosphate dehydrogenase (gapdh) gene and beta-actin was applied as a loading control. The Sirt1 deacetylase activity was expressed as percent relative to the LETO CON. Data are means ± SEM. (n = 4–5 per group). *p<0.05 vs CON.

In a previous report [30], we demonstrated that RGZ improves hepatic steatosis with a significant reduction of NAFLD activity score, serum, and liver lipids (FFA, TG, and total cholesterol) in rats. In addition, RGZ treatment up-regulated the gene expression of Adipoq, PPAR alpha (Ppara), Pparg, Sirt6, Ppargc1a, and Foxo1, and increased the protein levels of Sirt6, PGC-1α, phospho-Foxo1, phospho-LKB1, and phospho-AMPK. In this study, the gene expression of Sirt1 and its activity were measured to explore the effects of RGZ on hepatic Sirt1. RGZ treatment significantly up-regulated the gene expression and protein level of Sirt1 in both LETO and OLETF rat livers (Figure 1A and 1B). In addition, Sirt1 deacetylase activity was higher in RGZ-treated OLETF livers compared with that in untreated OLETF controls (Figure 1C).

### Effect of RGZ to reduce lipid accumulation and to activate the Sirt1/6-LKB1-AMPK pathway is abrogated by knockdown of Sirt1 and/or Sirt6 in AML12 mouse hepatocytes

**Figure 2:**
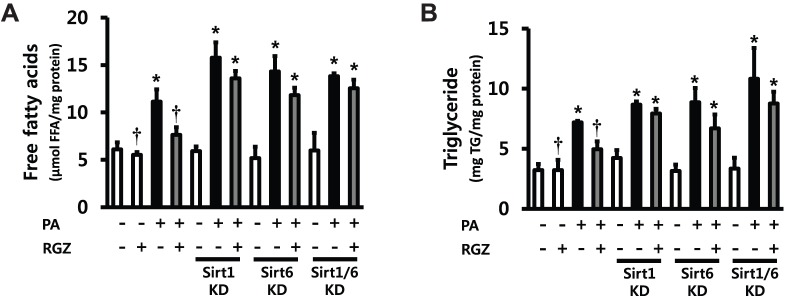
Knockdown (KD) of Sirt1 and/or Sirt6 diminished the effects of rosiglitazone (RGZ) to reduce hepatocyte lipid accumulation in AML12 mouse hepatocytes. Values of (A) free fatty acids (FFA) and (B) triglyceride (TG) were normalized with respect to protein concentrations. Data are means ± SEM. (n = 6 per group). *p<0.05 vs control (CON; white bar), †p<0.05 vs palmitic acid (PA) alone.

**Figure 3:**
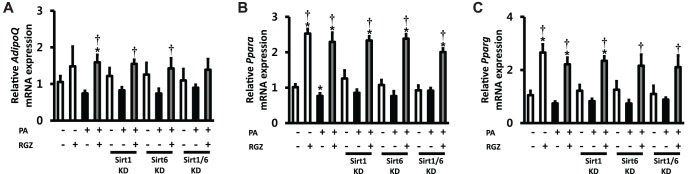
Knockdown (KD) of Sirt1 and/or Sirt6 suppressed the effects of rosiglitazone (RGZ) to regulate gene expression of (A) adiponectin (AdipoQ), (B) peroxisome proliferator-activated receptor alpha (Ppara), and (C) peroxisome proliferator-activated receptor gamma (Pparg). The expression levels were normalized with respect to those of the β-actin (Actb) gene. Data are means ± SEM. (n = 6 per group). *p<0.05 vs control (CON; white bar), †p<0.05 vs palmitic acid (PA) alone.

**Figure 4:**
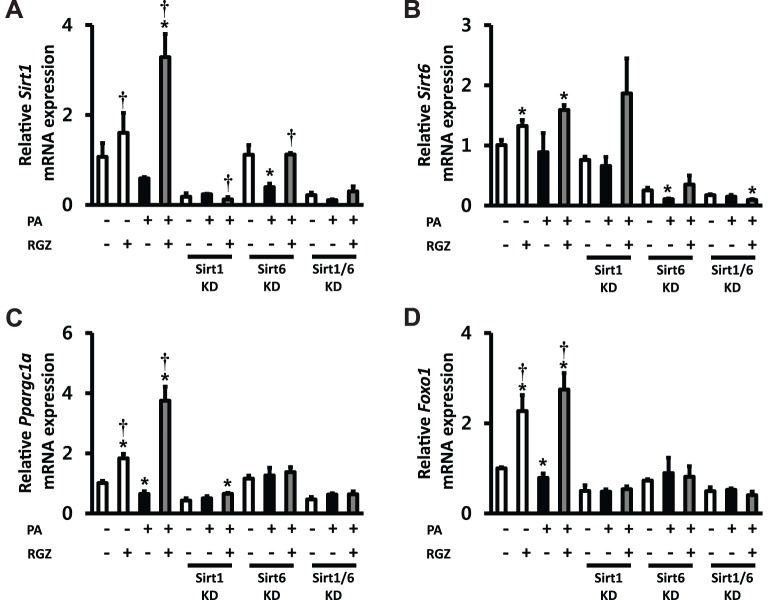
Knockdown (KD) of Sirt1 and/or Sirt6 suppressed the effects of rosiglitazone (RGZ) to regulate gene expression of Sirt1, Sirt6 and other related targets in AML12 mouse hepatocytes. The gene expression of (A) Sirt1, (B) Sirt6, (C) peroxisome proliferator-activated receptor gamma coactivtor-1-α (Ppargc1a), and (D) Forkhead box O1 (Foxo1). The expression levels were normalized with respect to those of the β-actin (Actb) gene. Data are means ± SEM. (n = 6 per group). *p<0.05 vs control (CON; white bar), †p<0.05 vs palmitic acid (PA) alone.

The findings from the *in vivo* experiment suggested that RGZ alters hepatic Sirt1 in a rodent model of NAFLD. A previous report from our research group has demonstrated that RGZ also upregulates Sirt6 and downstream target genes of Sirt1/6 (Ppargc1a and Foxo1) in the rat liver [30]. These findings suggest the possibility that Sirt1 and Sirt6 may have overlapped and synergistic effects in the action of RGZ. To seek further evidence for the functional relevance of Sirt1/6-stimulated AMPK activation to its regulation of hepatic lipid accumulation, RNAi-mediated gene silencing was conducted by transfecting AML12 cells with siRNA oligos targeting Sirt1 and/or Sirt6. Subsets of cells were incubated with PA for 48 h to induce hepatocyte steatosis, and then, PA and/or RGZ were administered for additional 24 h. PA incubation for 48 h induced hepatocyte steatosis with a significant increase in FFA and TG (Figure 2). As expected, RGZ treatment significantly reduced hepatocyte lipid accumulation. However, single and double knockdown targeting Sirt1 and Sirt6 diminished the effects of RGZ on hepatocyte lipid accumulation (Figure 2). RGZ treatment significantly upregulated the gene expression of PPAR alpha (Ppara), Pparg, Sirt1, Sirt6, Ppargc1a, and Foxo1 (Figures 3 and 4). Sirt1 and Sirt6 knockdown was confirmed by remarkably suppressed mRNA expression of Sirt1 and/or Sirt6, respectively (Figure 4A and B). Gene expression of AdipoQ, Ppara, and Pparg was up-regulated by RGZ and the up-regulation was unaltered by Sirt1 and/or Sirt6 knockdown, suggesting that those may act as up-stream regulators of Sirt1/6 (Figure 3). However, the RGZ-mediated up-regulation of Ppargc1a and Foxo1, major downstream targets of Sirt1/6, was suppressed or abolished by Sirt1 knockdown, which confirmed that Ppargc1a and Foxo1 are downstream regulators of Sirt1 in AML12 mouse hepatocytes (Figure 4C and D). Sirt6 knockdown affected the gene expression of Ppargc1a and Foxo1 to a lesser extent than Sirt1. Double-knockdown of Sirt1 and Sirt6 did not show significant synergistic effects on hepatocyte fat accumulation and the gene expression of Sirt1/6-related targets.

**Figure 5:**
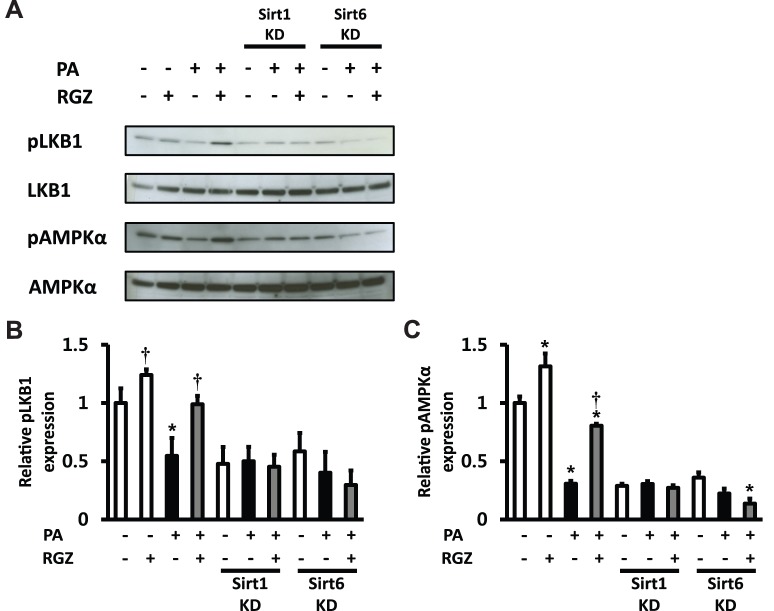
The effect of rosiglitazone (RGZ) on LKB1-AMPK pathway was abolished by knockdown (KD) of Sirt1 and/or Sirt6 in AML12 mouse hepatocytes. (A) Representative western blots for phosphorylated LKB1 at Ser428 (pLKB1) and phosphorylated AMPKα at Thr172 (pAMPKα) were shown and the expression of LKB1 and AMPKα was analyzed to confirm an equal protein loading control. Densitometric analysis of pLKB1 at Ser428 (B) and pAMPKα at Thr172 (C). Levels of phosphorylated AMPK and LKB were normalized to values for AMPK and LKB, respectively. Data are means ± SEM. (n = 6 per group). *p<0.05 vs control (CON; white bar), †p<0.05 vs palmitic acid (PA) alone.

Furthermore, whether the LKB1-AMPK pathway is altered following RGZ treatment and Sirt1 and/or Sirt6 knockdown was investigated in AML12 mouse hepatocytes. RGZ increased phosphorylation levels of LKB1 (Ser428) and AMPK (Thr172) in the presence of PA, and the knockdown of Sirt1 and Sirt6 suppressed the effects of RGZ on the LKB1/AMPK pathway (Figure 5), suggesting that Sirt1/6 regulates AMPK activation by altering LKB1 phosphorylation.

## Discussion

Sirtuins, NAD^+^-dependent deacetylases and ADP-ribosyltransferase, are implicated in the development of glucose and lipid metabolism-related diseases. However, the underlying mechanisms of how sirtuins are altered in the pathological conditions and how different isoforms of sirtuins interact are poorly understood. Here we show that RGZ alters hepatic Sirt1 in a rodent model of NAFLD and improves hepatocyte steatosis accompanied by elevations in adiponectin, Sirt1/6, and their downstream targets as well as AMPK phosphorylation *in vitro*. Another key finding in this study was that Sirt1 and Sir6 were not synergistic but compensatory factors that prevented hepatocyte steatosis based on the results of the Sirt1/6 single and double knockdown experiment.

The protective effects of RGZ and other TZDs against hepatic steatosis have been reported previously in human clinical trials [31]–[33]. Pioglitazone treatment reduces hepatic fat contents in type 2 diabetic subjects and ameliorates nonalcoholic steatohepatitis (NASH) accompanied by reduced blood levels of TG, alanine aminotransferase, and aspartate aminotransferase [31], [33], [34]. Similarly, RGZ improves hepatic steatosis and transaminase levels in NASH subjects [32]. Thus, the beneficial effect of RGZ against hepatic steatosis is supported by a number of findings; however, the underlying mechanisms remain inconclusive. The best characterized mechanism for TZD’s beneficial metabolic regulation is the adiponectin and Sirt-AMPK pathway [9], [12], [15], [18], [32], [35], [36]. Human clinical trials have reported that improvements in hepatic steatosis and NASH activity index score by TZDs in NASH subjects were associated with increased serum adiponectin levels [32], [36]. Involvement of the adiponectin-Sirt1-AMPK pathway in the RGZ-mediated protection against alcoholic fatty liver disease was demonstrated in ethanol-treated mice [18]. However, it has not yet been demonstrated in experimental models of NAFLD. In this study, 6 weeks of RGZ treatment upregulated Sirt1 expression and its activity in rat livers. This is the first evidence showing the RGZ-mediated alteration of hepatic Sirt1 in a rodent model of NAFLD. In previous reports from our group, the protective action of RGZ against hepatic steatosis is by coordinated regulation of adiponectin, Sirt6, and AMPK [30]. Up-regulation of Sirt1/6 and the downstream target genes of Sirt1/6 as well as activation of LKB1/AMPK by RGZ led us to hypothesize that RGZ may exert its positive effects by acting on both Sirt1 and Sirt6.

From these observations and accumulating evidence of sirtuins and their metabolic regulation, we speculate that Sirt1 and Sirt6 possess similarities in cellular localization and metabolic functions and question whether both sirtuins are synergistic or compensatory. To determine whether Sirt1 and Sirt6 are causally related to the development of hepatic steatosis and its amelioration by RGZ, and whether their actions are synergistic, we directly inhibited the Sirt1/6 pathway using RNAi-mediated gene silencing targeting Sirt1 and/or Sirt6 in AML12 mouse hepatocytes. Sirt1/6 knockdown aggravated hepatocyte fat accumulation as shown by increased TG content and suppressed the favorable effects of RGZ on hepatocyte steatosis. In addition, Sirt1/6 knockdown suppressed gene expression of Ppargc1a and Foxo1 as expected, and abolished the RGZ-mediated activation of LBK1 and AMPK. However, double knockdown of Sirt1 and Sirt6 did not result in a synergistic effect on the RGZ-mediated alterations. Conventionally, it has been considered that Sirt1 and Sirt6 exert their functions as NAD^+^-dependent deacetylase and ADP-ribosyltransferase. However, provocative reports have suggested that Sirt1 may be responsible for transcriptional regulation of downstream target genes [13], [37]. Sirt1 upregulated adiponectin gene expression in fully differentiated 3T3-L1 adipocytes by enhancing Foxo1 and C/enhancer-binding protein alpha interaction with the adiponectin promoter [37]. A positive correlation between Sirt1 and Sirt6 has been reported in mice, and, additionally, the increase in Sirt1 occurred earlier than that of Sirt6 in the mouse liver during fasting [13]. Additionally, whether Sirt1 is responsible for transcriptional regulation of Sirt6 was investigated, and the results showed that Sirt1 induced Sirt6 gene expression by forming a complex with Foxo3a and nuclear respiratory factor 1-binding sites on the Sirt6 promoter [13]. Based on these observations, we speculated that Sirt1 and Sirt6 may have synergistic effects on metabolic regulation. However, no synergistic effect between Sirt1 and Sirt6 was observed in this study, suggesting that the relationship between Sirt1 and Sirt6 may be compensatory as a back-up system.

Although we could not demonstrate the synergistic effects of two isoforms of sirtuins, our results suggest the possible interaction between Sirt1 and Sirt6. In normal AMP12 hepatocytes without PA treatment, Sirt1 mRNA expression was not altered by Sirt6 knockdown, but, Sirt6 mRNA expression tends to be lowered by Sirt1 knockdown. These results support the previous findings showing that Sirt1 is responsible for transcriptional regulation of Sirt6 [13]. In addition, regarding the effects on Ppargc1a and Foxo1, Sirt1 knockdown resulted in more profound suppression than Sirt6 knockdown. In a hepatocyte steatosis model with PA treatment, the regulatory effects of RGZ on Sirt1 were significantly blunted by Sirt6 knockdown, which suggests that up-regulation of Sirt1 by RGZ was partially dependent on Sirt6. On the other hand, RGZ’s effects on Sirt6 were not altered by Sirt1 knockdown in a hepatocyte steatosis model. At this point, we do not have plausible explanation except the possibility that RGZ’s action on Sirt1 and Sirt6 as well as interactions between Sirt1 and Sirt6 may be condition-specific (normal hepatocytes vs. steatic hepatocytes).

Despite some interesting findings in this study, our analyses had a limitation that part of the data regarding the involvement of Sirt1/6 in the RGZ’s effects was based on the alteration of mRNA expression. In general, the mRNA expression of specific target gene corresponds to its protein levels. However, it is possible to have a discrepancy between mRNA expression and protein levels due to post-transcriptional (e.g. mRNA stability), translational (e.g. initiation factor and trans-acting protein) or post-translational (e.g. proteolysis) regulations.

The present study was conducted to elucidate whether RGZ alters hepatic Sirt1 and to investigate the potential synergistic effects of Sirt1 and Sirt6 on regulation of hepatic fat accumulation by RGZ. Using a rat model of moderate obesity and insulin resistance and a cell model of hepatocyte steatosis, we report up-regulation of adiponectin, Sirt1/6, and downstream targets of Sirt1/6, and also, increases in both LKB1 and AMPK activities following RGZ treatment. Additionally, we demonstrated that Sirt1/6 knockdown abolished the effects of RGZ with regard to hepatocyte fat accumulation and the Sirt1/6-AMPK pathway, suggesting that Sirt1/6 regulates RGZ-mediated metabolic processes and that the relationship between Sirt 1 and Sirt6 may be compensatory without synergistic effects. These findings indicate that Sirt1 and Sirt6 are involved in the RGZ-mediated improvement of hepatocyte steatosis, and warrant future study to identify the interaction of different sirtuins on metabolic regulation.
